# Road Development and the Geography of Hunting by an Amazonian Indigenous Group: Consequences for Wildlife Conservation

**DOI:** 10.1371/journal.pone.0114916

**Published:** 2014-12-09

**Authors:** Santiago Espinosa, Lyn C. Branch, Rubén Cueva

**Affiliations:** 1 Department of Wildlife Ecology and Conservation, University of Florida, Gainesville, Florida, United States of America; 2 Escuela de Ciencias Biológicas, Pontificia Universidad Católica del Ecuador, Quito, Ecuador; 3 Wildlife Conservation Society - Ecuador Program, Quito, Ecuador; Universidade Federal do Acre (Federal University of Acre), Brazil

## Abstract

Protected areas are essential for conservation of wildlife populations. However, in the tropics there are two important factors that may interact to threaten this objective: 1) road development associated with large-scale resource extraction near or within protected areas; and 2) historical occupancy by traditional or indigenous groups that depend on wildlife for their survival. To manage wildlife populations in the tropics, it is critical to understand the effects of roads on the spatial extent of hunting and how wildlife is used. A geographical analysis can help us answer questions such as: How do roads affect spatial extent of hunting? How does market vicinity relate to local consumption and trade of bushmeat? How does vicinity to markets influence choice of game? A geographical analysis also can help evaluate the consequences of increased accessibility in landscapes that function as source-sink systems. We applied spatial analyses to evaluate the effects of increased landscape and market accessibility by road development on spatial extent of harvested areas and wildlife use by indigenous hunters. Our study was conducted in Yasuní Biosphere Reserve, Ecuador, which is impacted by road development for oil extraction, and inhabited by the Waorani indigenous group. Hunting activities were self-reported for 12–14 months and each kill was georeferenced. Presence of roads was associated with a two-fold increase of the extraction area. Rates of bushmeat extraction and trade were higher closer to markets than further away. Hunters located closer to markets concentrated their effort on large-bodied species. Our results clearly demonstrate that placing roads within protected areas can seriously reduce their capacity to sustain wildlife populations and potentially threaten livelihoods of indigenous groups who depend on these resources for their survival. Our results critically inform current policy debates regarding resource extraction and road building near or within protected areas.

## Introduction

Wildlife conservation within protected areas in the tropics is commonly challenged by two interacting factors: 1) protected areas are under pressure of large-scale resource extraction and associated road development, and 2) protected areas are often occupied by people who depend on wildlife for their survival [1], [2], [3]. In the Amazon Basin, protected areas and indigenous territories total 3,502,750 km^2^ representing 45% of the Basin [4]. This vast extension of protected forest could indicate a high probability of success for future conservation of wildlife populations in the region [5], [6], [7]. However, these lands are threatened by large-scale development, including road building to access valuable resources such as soils for agriculture, timber, hydropower, oil, gas and minerals [8], [9], [10], [11]. As national economies strongly depend on natural resources to develop, substantial increases in road networks within or adjacent to natural areas are predicted across the region and other tropical areas [10], [12], [13]. For example, plans have been developed for increasing roads within Ecuadorian protected areas and the Amazon Basin in Peru to gain access to oil and gas reserves [14], [15]. Increased accessibility to natural lands through road development negatively affects wildlife communities [16], [17], [18], [19]. Roads cause direct impacts on wildlife populations by providing access to hunters, introducing mortality through vehicle collisions, limiting animal dispersal, and altering other animal behavior [20], [21], [22], [23]. Additionally, roads initiate cascading effects by promoting colonization processes that lead to habitat loss, fragmentation and degradation [24].

More than 380 indigenous groups live scattered throughout Amazonia, including within protected areas [1], [4]. Many of these peoples persist in landscapes that have maintained high levels of biodiversity, including wildlife species that are important as a source of protein [25]. A variety of factors may have contributed to maintenance of harvested wildlife in these historically occupied lands, such as past low human population densities, lack of technologies allowing intense resource exploitation, subsistence economies that do not use wildlife as a commodity, or other cultural beliefs that favor conservation of biodiversity [26]. Additionally, indigenous hunters have been described as central-place foragers, meaning that they concentrate their activities near settlements [27], [28], [29], [30]. If settlement density remains low, and roads are not built within inhabited protected areas, a central-place forager behavior would facilitate wildlife persistence across the landscape through source-sink dynamics [31]. In a source-sink system, areas with high discrete rates of population increase for wildlife (i.e., λ>1), or sources for wildlife, are connected with areas where wildlife mortality is high (i.e., sinks, λ<1) through animal dispersal [32], [33], [34], [35]. If source areas are large enough, wildlife populations can persist in the sinks.

As roads are built throughout the Amazon Basin, factors that have contributed to maintenance of wildlife in these historically occupied lands are changing rapidly. By facilitating access to markets, roads promote commercial use of wildlife resulting in high pressure on wildlife populations [13], [36], [37]. Additionally, roads may significantly increase the portion of the landscape that is available for harvest and reduce the proportion that remains in source areas or wildlife refugia. Therefore, understanding the impacts of roads on wildlife harvest and trade from a geographical perspective is critical for informing national and international debates on permitting resource extraction in protected areas that currently contain human populations or will be colonized when roads are built [10], [14].

Important questions for wildlife management and conservation in face of increased road networks in Amazonia and other tropical regions are: How do roads affect spatial extent of hunting? How does market vicinity relate to local consumption and trade of bushmeat? How does market accessibility influence choice of game? To answer these questions, we examined hunting practices of the Waorani indigenous group in Yasuní Biosphere Reserve, located in the Amazon region of Ecuador. Yasuní is one of the most biodiverse areas on earth and is under severe pressure to allow increased road development to exploit oil reserves [14], [38]. Many other protected areas across the tropics are facing similar threats [3]. Our results and geographical analyses inform development policies associated with large-scale resource extraction in conservation landscapes inhabited by traditional or indigenous peoples.

## Methods

### Study Area

**Fig. 1:**
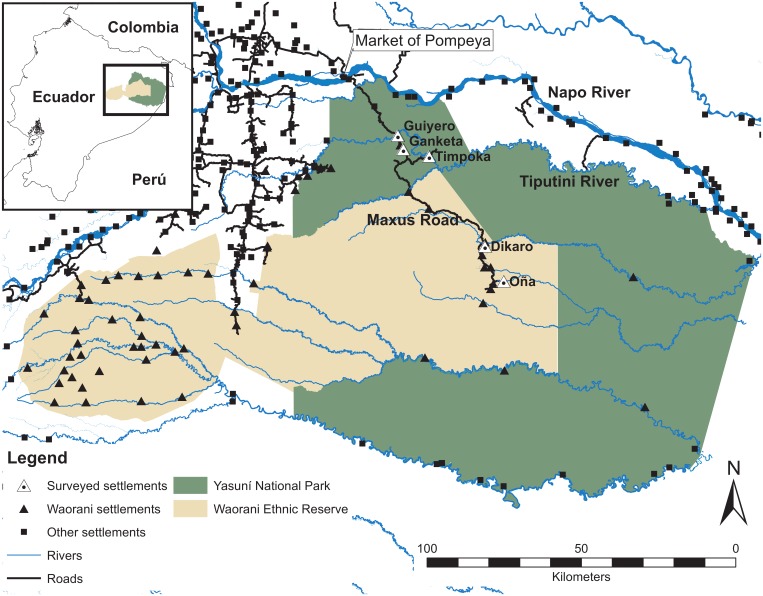
Study area and surveyed settlements along the Maxus Road in Yasuní Biosphere Reserve.

Yasuní Biosphere Reserve (hereafter Yasuní) covers an area of 18,000 km^2^ and is formed by Yasuní National Park and adjacent Waorani Ethnic Reserve (hereafter Waorani territory) (Fig. 1). Seasons in Yasuní are not clearly marked (mean monthly temperature, 22–34°C; annual rainfall, ∼3,000 mm) [39]. Vegetation is dominated by tall evergreen terra firme tropical forest, and flood plains and swamps occur along river margins [39].

Historically, Yasuní has been occupied by the Waorani indigenous group, which had a semi-nomadic lifestyle and economic system based on gathering and sharing of forest products and horticulture [40]. Bushmeat is the most important source of protein for Waorani, and hunting is an essential part of their culture. Waorani traditionally used spears to kill large prey, such as peccaries and tapir, and blowguns for arboreal species, such as monkeys or birds [41]. Prior to contact with western society, Waorani lacked canoes and feared large rivers; therefore, hunting was restricted to areas only accessible by foot [41]. In the early 1980s, the first road (Auca Road) was opened into the Waorani territory by Texaco Oil Company, and in the early 1990s, a 150-km road network was created by Maxus Oil Company within Yasuní. Nowadays, Waorani mostly have replaced blowguns and spears with the more effective shotguns [42]. Most Waorani now live in permanent settlements along rivers or the two roads in Yasuní.

Our study was located along Maxus Road, in an area inhabited by approximately 320 Waorani distributed in 11 settlements along the road’s margins. Access to Maxus Road is strictly controlled by the oil company operating in the area. Only indigenous groups can enter and move in the area freely. Waorani use free transportation provided by the oil company on a daily basis to reach hunting areas otherwise inaccessible and to travel every weekend to the closest market, Pompeya, a small town outside Yasuní (Fig. 1). This market provides an outlet for sale of wild game and for purchase of fuel for motors for dugout canoes and vehicles.

### Research Design and Data Collection

The following predictions formed the basis for our research design: 1) Roads significantly increase the proportion of landscape used by hunters. 2) Probability of hunting is higher closer to roads. 3) Hunters closer to markets harvest wildlife and sell more meat than hunters farther from markets. 4) Hunters who live closer to markets harvest a higher proportion of species that provide greater economic return in the bushmeat market (e.g., large-bodied species such as ungulates).

We surveyed hunting activities in three settlements close to markets and two settlements far from them (Fig. 1). Settlements close to markets included Guiyero [76°28′4.04″W, 0°36′44.1″S], Ganketa [76°27′6.41″W, 0°39′11.23″S] and Timpoka [76°22′35.58″W, 0°40′24.2″S], located at 32, 38 and 51 km from the market of Pompeya, respectively. To visit the market, inhabitants of these three settlements travel 1.5–2 hours. Guiyero was created with the establishment of the Maxus Road, between 1992 and 1993. In 2001 two families of Guiyero created Timpoka and later one of these two families moved to Ganketa. At the time of this study, Guiyero, Ganketa and Timpoka had populations of 35, six and 22 inhabitants organized in six, one and three households, respectively. The two settlements farther from markets included Dikaro [76°12′51.66″W, 0°56′13.6″S] and Oña [76°9′37.48″W, 1°2′22.27″S], which are located at 100 km and 120 km from the market, respectively. To visit the market, people from Dikaro and Oña travel 3.5–4.5 hours. Dikaro was created with road establishment in 1993 and is the largest settlement along the Maxus Road, with a population of 180 in 35 families. Oña comprises a single household of six people. We surveyed the entire population in all households except Dikaro, where we reached 144 people from 22 families. Our survey covered 67% of the Waorani population along Maxus Road.

Hunting can be influenced by other factors than accessibility and vicinity to markets, including biological (e.g., habitat quality, species distribution and abundance) and cultural factors (e.g., hunting techniques, taboos and hunting regulations) [43]. Alternative sources of cash for local people also could influence hunting practices [44], [45]. These factors could confound our observations if they varied across space in a way that correlated with distance of settlements from markets. However, this problem was unlikely. Vegetation cover throughout the entire study area is virtually intact. Deforestation is limited to areas where oil fields, settlements and roads are placed, and to small patches for swidden cultivation. Waorani in the five settlements share taboos and have equal hunting technologies. Hunting regulations do not exist. The only continuous source of jobs for Waorani along Maxus Road is the oil company. Waorani who want a job can get one for the same daily pay rate, and thus access to cash from employment does not vary spatially in our study area. At the time of our study, many Waorani did maintenance work for the oil company for 3–4 hours during the morning for 2–5 days per week and spent the rest of their time in other activities, including hunting. All Waorani settlements have similar access to transportation with the free bus service provided daily by the oil company along Maxus Road.

From January 2008 to April 2009, we obtained data on hunting activities of Waorani through self-reporting. We trained 15 Waorani assistants to report hunting activities on a daily basis within their households or households of related kin, and compensated each of them with US$ 30 per month. We visited settlements every 20–30 days to check completed questionnaires. As hunting is not an illegal activity for Waorani, participants were open to reporting their hunting activities. We monitored settlements close to markets for 14 months and settlements far from markets for 12 months.

Data collected for each animal killed included species, weight, weapon used, and use of each carcass (i.e., parts consumed or sold). Kill sites were marked on a map (scale 1∶200,000) printed on the data sheet for each kill. By accompanying Waorani on hunting trips or wildlife surveys, we observed that they had an extremely good sense of spatial location. Their accuracy in marking locations of kills on maps generally varied from 0–300 m when compared with locations obtained with a handheld GPS unit (Garmin GPSMAP 76Cx). Locations recorded on maps were digitized into a GIS database for analysis.

Animals were sold in parts (ungulates) or as ‘whole animal’ or ‘half animal’ (smaller game such as medium-sized rodents, monkeys or birds) in the Pompeya market. To estimate weight of bushmeat traded for ungulates sold in parts, we multiplied total weight of the harvested animal by percentages of body weight assigned to each part based on dressing yields of domestic pigs, cattle and white-tailed deer (*Odocoileus virginianus*) as follows: 8% of body weight for head, 14% for both front limbs, 24% for both hind limbs, 10% for both rib flanks, 15% for internal organs, and 19% for other parts including axial skeleton, abdomen and stomach contents [46], [47]. Middlemen immediately purchased all meat brought by Waorani to market. Generally all meat was sold for the same price per kilo, except meat from paca, which was more expensive because this species is preferred for its taste.

To conduct this study we obtained a permit from the Waorani Organization (NAWE) to work in their territory and a research permit from the Ministry of Environment of Ecuador to work in Yasuní National Park (012-IC-FA-PNY-RSO). Before the start of our study we conducted a meeting with each participating community to discuss our research objectives and to obtain their consent. Consent was obtained in an oral form rather than in writing based on advice from two Waorani leaders. Our research and oral informed consent protocol was approved by the University of Florida Institutional Review Board before research began (UFIRB #2007-U-36).

### Analytical Methods

#### Spatial extent of wildlife harvest

To evaluate effects of roads on spatial extent of hunting by Waorani, we used two approaches. First, we compared spatial extent of projected harvest area (km^2^) in the absence of the road with spatial extent of the observed hunting area. Second, we evaluated probability of hunting across the landscape as a function of sources of access (i.e., distance from roads, navigable rivers and settlements) and defined a harvest area based on this probability of hunting.

For our first approach we projected the accessible area prior to road building as the area within an 8-km radius from surveyed settlements. The 8-km distance is similar to the farthest distance hunters have been recorded to walk from a point of access in this study and in other areas of Amazonia (e.g., [17]). To estimate the current area harvested by Waorani, we used two methods, minimum convex polygon (MCP) which is the minimum area that contains all locations of kills and kernel density estimation [48]. We first used UTM locations of kills to estimate independent MCPs for the five settlements and subsequently merged these MCPs to obtain an overall harvested area. MCPs can overestimate the area used by hunters because unused areas may be present within the polygon. Therefore, we also estimated harvest area with kernel analysis using locations of kills and associated body mass to obtain cells representing amount of bushmeat extracted per unit of area and time (kg of bushmeat/km^2^/year). We performed kernel analysis using a spatial resolution of 250 m and smoothing factor of 8 km. MCP and Kernel analyses were conducted with software ESRI ArcGIS v10.

For our second approach we used logistic regression to assess probability of hunting as a function of accessibility based on observed kill sites (n = 2,997 UTM locations) and random points (n = 3,000) representing hypothetical unhunted, but accessible, sites. Random points were placed within an accessible area limited by: a) an 8-km buffer from roads or navigable rivers used by inhabitants of the five settlements, and b) a maximum distance of 40 km from settlements, which is similar to the maximum Euclidean distance Waorani traveled from their settlements to the farthest points of access along the Maxus Road and rivers, using either the bus provided by the oil company or motorized dugout canoes. Each observed kill site and random point was associated with three predictor variables representing accessibility: a) distance from road, b) distance from river, and c) distance from settlement where the hunter lived or nearest settlement from random points. We developed seven models that included all possible combinations of predictor variables and used AIC to select the best-fit model [49]. We assessed predictability performance of the best model with a threshold-independent receiver operating characteristic (ROC) curve [50]. We used the best model parameter estimates to plot probability of hunting across the landscape using the raster calculator tool in ArcGIS v10. For this purpose, we created three raster layers where centers of pixels (spatial resolution of 50 m) represented Euclidian distances to the nearest road, navigable river and settlement. We estimated harvest area based on probability of hunting by categorizing all pixels that had >20% probability of being hunted during our one-year study period as “hunted”.

#### Rates of bushmeat extraction and trade

We compared per capita daily extraction (kg of bushmeat/person/day) and trading rates of bushmeat (kg of bushmeat sold/person/day) between settlements near (Guiyero, Ganketa and Timpoka) and far (Dikaro and Oña) from markets. We estimated per capita daily extraction and trading rates for each surveyed month with data grouped for settlements close and far from markets. We used a Wilcoxon signed rank test to compare extraction and trading rates for 12 consecutive months between the two groups.

#### Differential use of game at varying distance from market

We evaluated differences in composition of taxa harvested by hunters as a function of distance from market. For this purpose, we identified species that were most important for bushmeat trade (i.e., species with high market value) and calculated the proportion of the total number of animals killed by each household that corresponded to these species. A species was considered of high bushmeat market value if: 1) at least 100 kg were harvested, and 2) greater than 20% of the species’ total harvested biomass was traded (see S1 Appendix for total biomass harvested and percent of biomass that was traded, WST, for all species). We then calculated proportion of the total number of animals killed by each household that corresponded to species of high market value and compared these proportions for households near (n = 10) and far (n = 23) from markets using a Wilcoxon rank sum test.

## Results

A total of 3,101 animals (53,700 kg) representing at least 51 species (24 birds, 23 mammals and four reptiles) were harvested between January 2008 and April 2009 by 33 Waorani households (Table 1; S1 Appendix: full account of species harvested). Four of the five species of ungulates present in the area, white-lipped peccary (*Tayassu pecari*), collared peccary (*Pecari tajacu*), red brocket (*Mazama americana*) and South American tapir (*Tapirus terrestris*), were the most important source of bushmeat, contributing 89% of total harvested biomass (Table 1). White-lipped and collared peccaries, the most intensively hunted species, accounted for 65% of total biomass extracted and 75% of total biomass traded (Table 1). Approximately, 35% of total biomass of harvested game was commercialized.

**Table 1:** Most important species harvested and traded as bushmeat by Waorani along Maxus Road in Yasuní.

	Harvest			Trade		
Species^a^	n	kg	PTB^b^	kg	WST^c^	PTT^d^
White-lipped peccary (*Tayassu pecari*)	975	26,493	49.3	10,510	39.7	56.7
Collared peccary (*Pecari tajacu*)	448	8,498	15.8	3,316	39.0	17.9
South American Tapir (*Tapirus terrestris*)	58	8,200	15.3	2,166	26.4	11.7
Red brocket (*Mazama americana*)	155	4,517	8.4	1,524	33.7	8.2
Amazonian brown brocket (*Mazama nemorivaga*)	11	162	0.3	18	11.4	0.1
Woolly monkey (*Lagothrix poeppigii*)	280	1,838	3.4	249	13.6	1.3
Spider monkey (*Ateles belzebuth*)	73	556	1.0	61	10.9	0.3
Howler monkey (*Alouatta seniculus*)	51	340	0.6	33	9.8	0.2
Lowland paca (*Cuniculus paca*)	117	1,032	1.9	335	32.4	1.8
Black agouti (*Dasyprocta fuliginosa*)	49	238	0.4	54	22.6	0.3
Salvin’s curassow (*Mitu salvini*)	173	693	1.3	60	8.6	0.3
Spix’s guan (*Penelope jacquacu*)	155	165	0.3	3	1.6	0.0
Blue-throated piping-guan (*Pipile cumanensis*)	102	104	0.2	2	2.0	0.0
Yellow-footed tortoise (*Chelonoidis denticulata*)	31	169	0.3	110	65.4	0.6
Other species (37)	423	688	1.3	91	13.2	0.5
Total	3,101	53,693	100.0	18,532		100.0

a Only species that have a total harvest above 100 kg are listed;

b Proportion of Total Biomass: Percentage of species contribution to total biomass harvested;

c Within Species Trade: Percentage of species’ biomass harvested that is traded;

d Proportion of Total Trade: Percentage of species contribution to total biomass traded.

### Roads and Spatial Extent of Wildlife Harvest

**Fig. 2:**
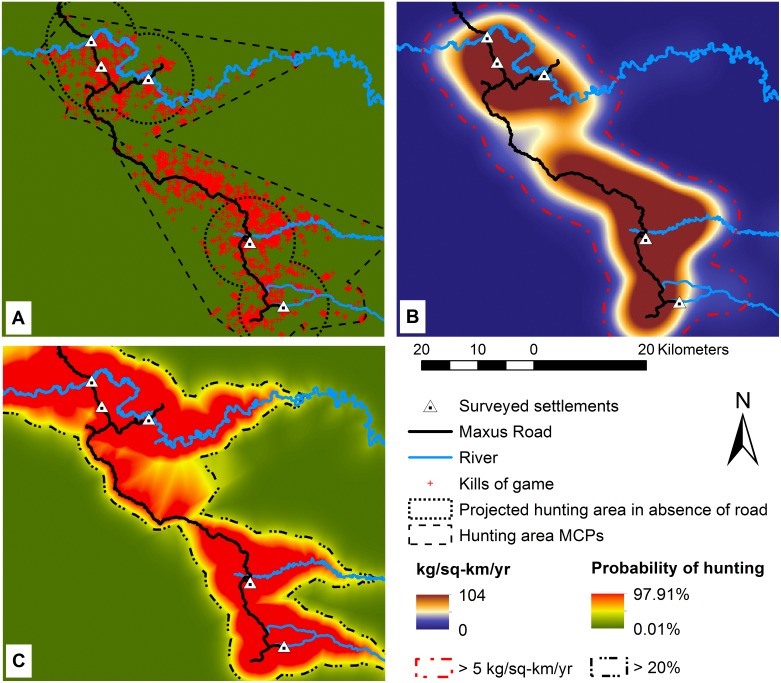
Observed hunting area for five Waorani settlements along the Maxus Road in Yasuní. Hunting area estimated by using: (A) Minimum Convex Polygons, (B) extraction rate (kg/km^2^/yr), and (C) probability of hunting based on landscape accessibility.

Maxus Road led to a significant increase in spatial extent of hunting in Yasuní. The MCPs of hunting areas for the settlements of Guiyero, Ganketa, Timpoka, Dikaro and Oña were 640, 275, 378, 925 and 138 km^2^, respectively. When combining MCPs of the five settlements, the total harvested area was 1,616 km^2^, which doubles the projected accessible area (790 km^2^) in the absence of roads (Fig. 2A). The maximum Euclidean distance walked from a point of access (i.e., road or river) to a kill site by Waorani hunters was 7 km (mean = 1.36, SD = 1.18, n = 2,997 UTM locations of animals hunted), but only 37 hunting records were at a distance greater than 5 km. The maximum Euclidean distance hunters traveled from their settlements to sites where animals were hunted was 37 km (mean = 9.45 km, SD = 5.95 km, n = 2,997).

Kernel analysis provided estimates of biomass extraction that varied from 0–104 kg/km^2^/yr. Higher extraction rates occurred along roads and rivers (Fig. 2B). Based on the sum of all pixels with an extraction rate of at least 5 kg/km^2^/yr, the total harvested area was 1,560 km^2^ (Fig. 2B). Hunters reached otherwise inaccessible areas by moving along rivers in motorized dugout canoes with fuel purchased at local markets or obtained from the oil company and along Maxus Road using the transportation system provided by the oil company, hitchhiking, or their own vehicles.

The best model in predicting probability of hunting incorporated the three predictor variables (distances of kill sites from hunters’ settlements, nearest navigable rivers and Maxus Road) and had very good discrimination capacity (area under the ROC curve = 0.92, S2 Appendix) [51]. No other candidate model was competitive (second best model ΔAIC = 779, AIC_w_<0.01%, S3 Appendix). Based on this model, annual probability of hunting was 20% or more in an area of 1,684 km^2^ (Fig. 2C). Hunting was less likely to occur at distances farther from rivers and roads, but increased with distance from settlements (Table 2).

**Table 2:** Untransformed parameter estimates of best-fit model to estimate probability of hunting as a function of landscape accessibility.

			Likelihood Ratio		
Parameter	β	SE	Wald*-X^2^*	df	*p*
Intercept	2.682	0.087	953	1	0.000
Road	−0.518	0.016	1017	1	0.000
River	−0.652	0.022	895	1	0.000
Settlement	0.323	0.015	468	1	0.000

Pseudo R^2^ = 0.496.

### Extraction and Trading Rates of Game

The median bushmeat extraction rate in settlements close to market was 0.75 kg/person/day (range = 0.44–1.29), and in settlements farther away extraction rate was 0.53 kg/person/day (range = 0.31–0.88; Z = 2.118, p = 0.034). The median amount of bushmeat traded by settlements close to markets was 0.26 kg/person/day (range = 0.17–0.66). Those farther from markets traded a median of 0.18 kg/person/day (range = 0.09–0.39; Z = 2.353, p = 0.016). Bushmeat kept for self-consumption did not differ between Waorani close and far from markets (median_close_ = 0.49 kg/person/day, range = 0.19–0.96; median_far_ = 0.36 kg/person/day, range = 0.19–0.67; Z = 0.941, p = 0.380).

### Differential Use of Game Species

Seven species were frequently commercialized (i.e., had high market value): white-lipped peccary, collared peccary, red brocket, South American tapir, paca (*Cuniculus paca*), agouti (*Dasyprocta fuliginosa*) and yellow-footed tortoise (*Chelonoidis denticulata*). For these seven species, more than 22% of their harvested biomass was sold in the market, and the number of individuals harvested ranged from 31 yellow-footed tortoises to 975 white-lipped peccaries (Table 1, S1 Appendix). Large primates and large terrestrial birds were commercialized less frequently although they contributed substantial biomass to total harvest (Table 1, S1 Appendix).

**Fig. 3:**
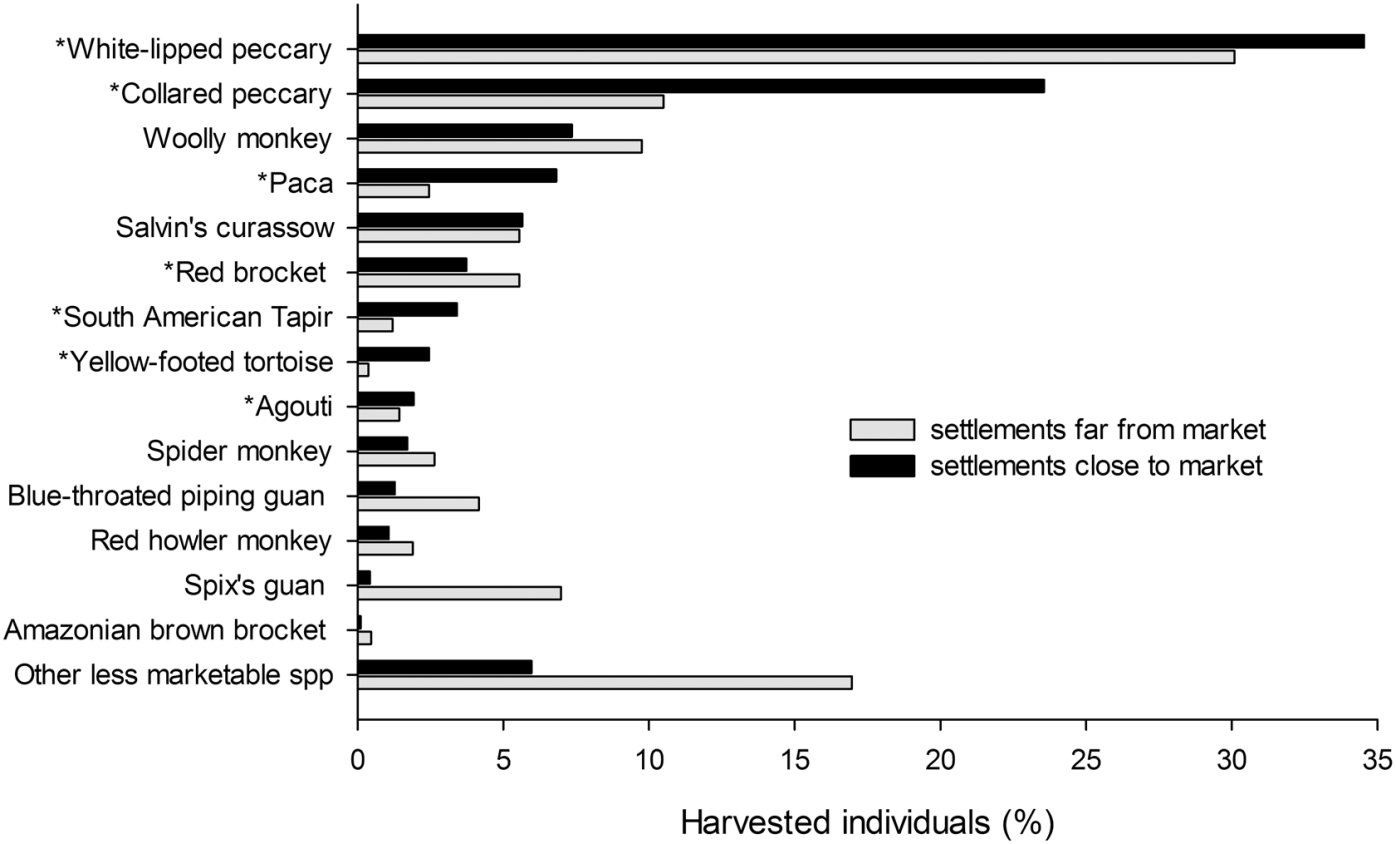
Contribution of individuals from the 14 most hunted species to the total kills at settlements close (n = 938 animals) and far (n = 2163 animals) from markets. *Most commercialized game species.

Harvests of households close to markets (n = 10) comprised a higher proportion of individuals of species that have high commercial value than harvests of hunters far away (n = 23) from markets (median_close_ = 79%, range = 54–100%; median_far_ = 47%, range = 26–89%; W = 193, p = 0.002). For six out of seven species with high value in the bushmeat market, individuals of these species comprised a higher proportion of the total harvest close to markets than farther from markets (Fig. 3). The exception was red brocket, which was hunted more in areas farther from market. Collared peccaries presented the largest difference comprising 25% of total individuals hunted by households close to markets, but only 10% of individuals harvested far from markets (Fig. 3). Less commercialized species were harvested in lower proportions close to markets than farther from them, with the exception of Salvin’s curassow (*Mitu salvini*), which was harvested equally by both groups of hunters (Fig. 3).

## Discussion

A spatially explicit approach to evaluation of hunting provides a means to visualize and to better understand the consequences of road creation within human-inhabited protected areas for wildlife conservation and game management. We evaluated catchment area with two different approaches not previously used in related studies: kernel analysis to estimate annual bushmeat extraction per unit of area, and logistic regression using spatially explicit data to estimate probability of hunting as a function of distance from roads, rivers and settlements. Rates of bushmeat extraction were higher near sources of access, and probability of hunting decreased with distance from roads or rivers and increased with distances from villages. These patterns reflect the increased effort required by hunters to walk further distances and local game depletion near settlements, respectively [17], [27], [29], [30], [51], [52]. Our approach, which combines local participation in monitoring and spatially explicit modeling, can be particularly useful for wildlife management in the tropics for several reasons: 1) it allows accurate identification of catchment areas; 2) information can be easily used to model possible effects of new road development on wildlife conservation; and 3) as data were recorded by local people, study outcomes can be easily used to develop community-based wildlife management plans.

### Roads and Increased Spatial Extent of Hunting

Although subsistence hunters in the Neotropics traditionally behaved as central-place foragers and acquired most of their game near settlements [27], [28], [29], Waorani no longer follow this pattern when roads and markets are available, but rather extend hunting to areas not accessible by foot (e.g., 10–40 km from settlements). Waorani surveyed along the Maxus Road harvested an area of 1,600 km^2^, which is twice the projected harvest area around these settlements in the absence of roads. By increasing spatial extent of hunting and making large areas permanently accessible to hunting, roads can threaten the persistence or ecological functionality of game populations within inhabited protected areas. In contrast to their current permanent settlements along roads, historically the Waorani were semi-nomadic, and therefore their hunting areas moved through time across the landscape, but always were limited to areas they could reach on foot [41]. Other indigenous groups, such as the Yanomami in Brazil, still maintain hunting campsites away from the villages or move the villages periodically [53]. This more mobile pattern of hunting results in use of small areas (i.e., within walking distance of hunting camps or villages) for discrete periods of time, within a larger unhunted area that serves as a wildlife refugium [53]. The spatial and temporal patterns of this hunting strongly contrasts with hunting patterns associated with Waorani communities in permanent settlements along roads. On a daily basis, Waorani hunters move from their settlements up to 40 km along roads to start hunting, and the area along the road is accessible to hunting permanently.

Despite the increase in the area hunted on a permanent basis after road building, Waorani along Maxus Road have continued to extract significant amounts of bushmeat, including vulnerable species (e.g., this study, [54], [55]). A previous study on Maxus Road used Robinson and Redford’s harvest model to assess harvest sustainability and concluded that extraction rates of large monkeys and white-lipped peccaries were above sustainable levels [56], [57]. At the time of our study, 5 years later, these species continued to be harvested at even higher rates. With our spatial analyses, we observe that this intense bushmeat extraction likely persists because Maxus Road is within a vast area of intact forest functioning as a wildlife refugium that supplements wildlife in hunted areas. Similarly, despite being extracted above sustainable levels, the South American tapir, a species highly sensitive to hunting, has been documented in harvested areas in other parts of its geographic range when these areas were adjacent to sources [31], [58]. In contrast, in the western portion of Yasuní where road and settlement density are higher, tapirs are scarce and white-lipped peccaries have not been reported in the last 15 years [42], [59].

For wildlife to persist, harvest must be managed (e.g., by establishing and enforcing hunting quotas) or large areas must remain inaccessible to hunters in order to serve as refugia [31], [32], [33], [60]. Because roads increase accessibility, roads impact conservation landscapes that function as source-sink systems. For example, in northern Congo, the two strongest predictors for elephant density are distance to Nouabalé-Ndoki National Park (i.e., elephant numbers decrease with distance from park) and distance to roads in adjacent logging concessions where elephants are poached (i.e., elephant numbers increase with distance from roads) [61]. To maintain viable wildlife populations in a source-sink system, a significant area must be maintained as a wildlife refuge. In our study site, a single road segment of 117 km made an area accessible to hunters that corresponds to 9% Yasuní National Park and Waorani Ethnic Reserve. This is a significant proportion of the conservation area. For example, to avoid population decline of slow-reproducing species in the neighboring Peruvian Amazon, such as South American tapir and spider monkey (*Ateles paniscus*), at least 63% or 72% of the area, respectively, must be maintained as a protected area with no hunting to act as a source to offset mortality from hunting [31]. Yasuní is accessible to hunters through other roads and rivers, in addition to the Maxus Road, and a new road is currently under construction into its core area, representing a significant threat to future of species sensitive to hunting [62].

### Extraction and Trading Rates

Commercial hunting increases as settlement distance to markets decreases, and roads stimulate trade of wildlife by increasing accessibility to markets [13], [37], [63], [64], [65]. Bushmeat extraction and trade from Waorani hunters along the Maxus Road has increased significantly throughout the last decade. In 2002 settlements of Guiyero, Timpoka and Dikaro extracted 6,360 kg of bushmeat in a five-month period, and less than 4% of this harvest was traded at the market of Pompeya [54]. By 2007 annual bushmeat trade in Pompeya was estimated at 10,500 kg/year with Waorani from these same settlements contributing nearly half of this biomass [55]. In our study, Waorani from Guiyero, Timpoka, Dikaro and two additional households provided close to 18,500 kg of bushmeat for trade in the same market in a single year. Although methodological differences exist between the three studies, bushmeat extraction for commercial purposes along Maxus Road clearly increased rapidly between 2002 and 2009. This increase in bushmeat trade is not linked to population growth in these communities. The number of Waorani in these communities increased by only 7.7% during the same period.

At the time of this study trade of bushmeat was illegal in Ecuador, but there was no law enforcement and bushmeat transactions occurred freely in the market. The price of bushmeat at the market in the town of Pompeya was US$3–6/kg, similar to the value of poultry and beef at grocery stores in cities. However, the price of bushmeat can increase significantly with distance from catchment areas [66]. For example, the price of bushmeat in the neighboring city of Lago Agrio was close to 50% higher than the price of meat from domestic animals, indicating a good market for this resource [55]. Currently, the Ministry of Environment, in collaboration with other institutions such as Ecuador’s Environmental Police Unit, is controlling wildlife traffic outside protected areas. However, although bushmeat trade can no longer be observed in local markets, clandestine trade by the Waorani occurs (SE personal observations) and likely will continue as long as a strong market remains for bushmeat.

Even though Waorani along Maxus Road are using a significant amount of bushmeat for trade, they currently maintain appropriate levels of meat consumption. Other studies estimate Neotropical hunters consume 65–70% of an animal’s live weight and that healthy consumption of meat is about 0.25 kg/person/day [26], [41], [67]. Three decades ago Waorani consumed 0.28 kg/person/day and, in our study, about 0.30–0.32 kg/person/day after correcting for the edible proportion of game [41]. Importantly, the amount destined for self-consumption did not differ between Waorani close to and far from markets. However, if access to Yasuní and human populations continue to grow with new road development, not only may game populations decline but also the future ability of Waorani to procure their source of protein could be compromised.

### Differential Use of Game

Hunting involves the harvest of animal populations, commonly for meat consumption, but also for other uses such as medicine, trophies or live animals used as pets or for trade [68]. Choice of game will depend on factors such as animal abundance, animal behavior, hunting technologies, taste preferences, taboos or community regulations [43], [69], [70]. Also, choice of game will be related to access and nature of available markets. For example, if there is a demand for bushmeat, harvest of species that are preferred by consumers, or species that provide the greatest financial return, will increase [13], [51], [71], [72]. Access to markets has led to significant increases in the proportion of large-bodied game harvested by Waorani, principally peccaries, which are among the most important terrestrial game for Amazonian hunters [26], [43], [51], [58], [73]. Because the price per kilogram of bushmeat in the markets near our study area is similar for all game species (except the highly preferred paca), large-bodied animals provide a higher return per individual than smaller animals. The only ungulates that were not hunted in a higher proportion near markets were red brocket and Amazonian brown brocket (*M. nemorivaga)*. These species also are present in low numbers in other hunting studies (e.g., 6 out of 3,165 kills [41], 50 out of 2,355 kills [42], 28 out of 3,004 kills [52]). This low harvest may reflect the difficulty of hunting these deer, which move very fast and quietly through the forest.

By intensively targeting large-bodied organisms, hunters may induce shifts in species composition that affect structure and function of Neotropical forest systems [74]. Peccaries and tapirs are important for dispersal of palm seeds and other tropical plant species that produce large fruits [75], [76]. Peccaries also are important ecosystem engineers for anurans because they create wallows where tadpoles can develop [77]. Additionally, peccaries are generally the most important prey for jaguar (*Panthera onca*), the top predator in Neotropical terrestrial systems [78]. Decreases in peccary populations could reduce abundance of jaguars and lead to changes in top-down ecosystem processes, as has been noted with loss of top predators in other systems [79], [80], [81], [82].

### Management Implications

Our results reveal the importance of careful evaluation before constructing new roads within inhabited protected areas. Given the historical lack of resources to manage protected areas in the tropics, preserving wildlife refugia may be the most secure option for conserving wildlife in the long term if these areas are large enough to compensate for high mortality of large-bodied organisms within accessible areas. However, this option increasingly is compromised by expanding road networks. To conserve the biodiversity of Yasuní and maintain wildlife harvest by Waorani, an adequate proportion of Yasuní needs to remain inaccessible to hunting, and at the same time, additional programs for wildlife management need to be developed. Current new road development within Yasuní to expand oil extraction compromises the reserve’s capacity to sustain wildlife populations and commercial use of game in accessible areas. In face of changes induced by roads, strategies for harvest management need to be investigated and implemented, such as community-based programs that address long-term harvest of game by Waorani without threatening the integrity of the ecosystem.

An argument could be made that roads created for resource extraction within protected areas should not lead to ecosystem destruction if access is properly regulated. For example, Rabi oil concession operated by multinational Shell in Gabon applied strict control to impede hunting along roads within the concession. As a result, wildlife abundance was higher inside the concession than in surrounding unprotected areas where road density was higher [19]. However, control of hunting or other problems related to access (e.g., colonization) along roads by private companies involved in resource extraction is not likely to be a long-term solution in Yasuní or most regions. For example, oil concessions frequently change ownership, and emphasis placed on protecting the environment varies widely among companies [19]. Furthermore, when resource extraction activities are terminated, roads remain. As revenues from extractive activities, such as oil or mining, decrease or disappear, no resources are available to enforce control for illegal hunting, colonization and associated habitat loss, fragmentation and degradation. This is especially true in tropical protected areas, generally located within low-income countries, where pressure on land and natural resources are high, and where environmental institutions are weak and resources available for biodiversity conservation are limited [83], [84].

The extent of landscape accessibility as a function of road density will depend on a myriad of factors, such as spatial distribution of roads and rivers and topography. However, we expect roads to have a large impact on accessibility in Amazonia because roads will provide access to extensive river networks. Also, the relatively flat topography of this region facilitates road building. Our analyses only considered one set of processes associated with roads within natural areas–hunting and market accessibility. Other processes facilitated by roads such as colonization, deforestation, risk of fires, and introduction of disease and invasive species bring additional threats to the integrity of these high diversity ecosystems, and further highlight the need to make roads a central point of discussion in any development plans [85], [86], [87], [88], [89].

## Supporting Information

S1 Appendix **Full account of species harvested by Waorani along Maxus Road in Yasuní Biosphere Reserve, Ecuador.**
(DOCX)

S2 Appendix **Threshold-independent receiver operating characteristic (ROC) curve to test predictability performance of model to estimate probability of hunting.**
(DOCX)

S3 Appendix **Selection of best-fit model with Akaike Information Criterion (AIC).**
(DOCX)
