# Supplementary material for: Road Development and the Geography of Hunting by an Amazonian Indigenous Group: Consequences for Wildlife Conservation
Source: PLoS One. 2014 Dec 9;9(12):e114916. doi: 10.1371/journal.pone.0114916 (PMC4260950; doi:10.1371/journal.pone.0114916)
Supplement: S1 Appendix — Full account of species harvested by Waorani along Maxus Road in Yasuní Biosphere Reserve, Ecuador. (DOCX) [file pone.0114916.s001.docx]

Appendix S1. Full account of species harvested by Waorani along Maxus Road in Yasuní Biosphere Reserve, Ecuador.

| **Species** | **Dikaro**  **(n)** | **Oña**  **(n)** | **Guiyero**  **(n)** | **Ganketa**  **(n)** | **Timpoka**  **(n)** | **Total Harvest** | | | **Total Trade** | | |
| --- | --- | --- | --- | --- | --- | --- | --- | --- | --- | --- | --- |
|  |  |  |  |  |  | **n** | **kg** | **PTB^a^** | **(kg)** | **WST^b^** | **PTT^c^** |
| **AVES** | 588 | 58 | 51 | 11 | 43 | 751 | 1,178.7 | 2.2 | 72.9 | 6.2 | 0.4 |
| **Columbiformes** | 3 | - | 0 | 1 | - | 4 | 0.6 | 0.0 | 0.0 | 0.0 | 0.0 |
| *Columba* sp. | - | - | - | 1 | - | 1 | 0.1 | 0.0 | 0.0 | 0.0 | 0.0 |
| *Leptotila rufaxilla* | 3 | - | - | - | - | 3 | 0.5 | 0.0 | 0.0 | 0.0 | 0.0 |
| **Galliformes** | 338 | 26 | 41 | 5 | 26 | 436 | 968.9 | 1.8 | 65.4 | 6.7 | 0.4 |
| *Mitu salvini* | 114 | 6 | 31 | 2 | 20 | 173 | 692.5 | 1.3 | 59.8 | 8.6 | 0.3 |
| *Nothocrax urumutum* | - | 1 | 2 | - | 1 | 4 | 5.4 | 0.0 | 0.9 | 16.7 | 0.0 |
| *Odontophorus gujanensis* | - | 2 | - | - | - | 2 | 1.8 | 0.0 | 0.4 | 19.5 | 0.0 |
| *Penelope jacquacu* | 139 | 12 | 3 | 1 | - | 155 | 165.3 | 0.3 | 2.3 | 1.4 | 0.0 |
| *Pipile cumanensis* | 85 | 5 | 5 | 2 | 5 | 102 | 103.9 | 0.2 | 2.1 | 2.0 | 0.0 |
| **Gruiformes** | 27 | 3 | 4 | 1 | - | 35 | 29.3 | 0.1 | 3.6 | 12.4 | 0.0 |
| *Psophia crepitans* | 27 | 3 | 4 | 1 | - | 35 | 29.3 | 0.1 | 3.6 | 12.4 | 0.0 |
| **Passeriformes** | 2 | 1 | - | 2 | 4 | 9 | 1.5 | 0.0 | 0.0 | 0.0 | 0.0 |
| *Clypicterus oseryi* | - | - | - | - | 4 | 4 | 0.7 | 0.0 | 0.0 | 0.0 | 0.0 |
| *Cyanocorax violaceus* | - | 1 | - | - | - | 1 | 0.2 | 0.0 | 0.0 | 0.0 | 0.0 |
| *Formicarius* sp. | - | - | - | 1 | - | 1 | 0.1 | 0.0 | 0.0 | 0.0 | 0.0 |
| *Myiarchus tuberculifer* | - | - | - | 1 | - | 1 | 0.1 | 0.0 | 0.0 | 0.0 | 0.0 |
| *Psarocolius angustifrons* | 2 | - | - | - | - | 2 | 0.4 | 0.0 | 0.0 | 0.0 | 0.0 |
| **Piciformes** | 120 | 22 | 2 | 1 | 9 | 154 | 66.8 | 0.1 | 2.8 | 4.2 | 0.0 |
| *Campephilus rubricollis* | 1 | - | - | - | - | 1 | 0.5 | 0.0 | 0.0 | 0.0 | 0.0 |
| *Pteroglossus pluricinctus* | 1 | 1 | - | - | 1 | 3 | 1.4 | 0.0 | 0.0 | 0.0 | 0.0 |
| *Ramphastos* sp*.* | 14 | 2 | - | 1 | - | 17 | 8.8 | 0.0 | 0.7 | 7.7 | 0.0 |
| *Ramphastos tucanus* | 104 | 19 | 2 | - | 8 | 133 | 56.1 | 0.1 | 2.1 | 3.7 | 0.0 |
| **Psittaciformes** | 61 | 5 | 2 | 1 | 3 | 72 | 59.5 | 0.1 | 1.1 | 1.9 | 0.0 |
| *Amazona farinosa* | 8 | - | - | 1 | 1 | 10 | 6.4 | 0.0 | 0.0 | 0.0 | 0.0 |
| *Ara ararauna* | 8 | 1 | 1 | - | 1 | 11 | 11.8 | 0.0 | 0.2 | 1.7 | 0.0 |
| *Ara macao* | 38 | 4 | - | - | - | 42 | 38.2 | 0.1 | 0.9 | 2.4 | 0.0 |
| *Ara* sp. | 2 | - | - | - | - | 2 | 1.8 | 0.0 | 0.0 | 0.0 | 0.0 |
| *Brotogeris cyanoptera* | - | - | - | - | 1 | 1 | 0.1 | 0.0 | 0.0 | 0.0 | 0.0 |
| *Pyrilia barrabandi* | 1 | - | - | - | - | 1 | 0.2 | 0.0 | 0.0 | 0.0 | 0.0 |
| *Pionus menstruus* | 4 | - | 1 | - | - | 5 | 0.9 | 0.0 | 0.0 | 0.0 | 0.0 |
| **Tinamiformes** | 37 | 1 | 2 | - | 1 | 41 | 52.1 | 0.1 | 0.0 | 0.0 | 0.0 |
| *Crypturellus cinereus* | 18 | - | - | - | - | 18 | 22.4 | 0.0 | 0.0 | 0.0 | 0.0 |
| *Crypturellus* sp. | 4 | - | 1 | - | - | 5 | 5.2 | 0.0 | 0.0 | 0.0 | 0.0 |
| *Tinamus major* | 15 | 1 | 1 | - | 1 | 18 | 24.5 | 0.0 | 0.0 | 0.0 | 0.0 |
| **MAMMALIA** | 1,400 | 104 | 560 | 43 | 201 | 2,308 | 52,251.6 | 97.3 | 18,330.2 | 35.1 | 98.9 |
| **Artiodactyla** | 972 | 36 | 404 | 28 | 149 | 1,589 | 39,670.0 | 73.9 | 15,369.0 | 38.7 | 82.9 |
| *Mazama americana* | 112 | 8 | 18 | 3 | 14 | 155 | 4,516.9 | 8.4 | 1,524.4 | 33.7 | 8.2 |
| *Mazama nemorivaga* | 10 | - | - | - | 1 | 11 | 162.0 | 0.3 | 18.5 | 11.4 | 0.1 |
| *Pecari tajacu* | 214 | 13 | 148 | 11 | 62 | 448 | 8,497.8 | 15.8 | 3,315.6 | 39.0 | 17.9 |
| *Tayassu pecari* | 636 | 15 | 238 | 14 | 72 | 975 | 26,493.3 | 49.3 | 10,510.5 | 39.7 | 56.7 |
| **Carnivora** | 9 | 10 | 5 | - | 2 | 26 | 177.0 | 0.3 | 19.8 | 11.2 | 0.1 |
| *Eira barbara* | - | 1 | - | - | - | 1 | 3.0 | 0.0 | 0.0 | 0.0 | 0.0 |
| *Nasua nasua* | 8 | 5 | 5 | - | 1 | 19 | 94.1 | 0.2 | 14.9 | 15.9 | 0.1 |
| *Panthera onca* | - | 1 | - | - | 1 | 2 | 72.5 | 0.1 | 0.9 | 1.2 | 0.0 |
| *Potos flavus* | 1 | 3 | - | - | - | 4 | 7.4 | 0.0 | 4.0 | 54.3 | 0.0 |
| **Cingulata** | 3 | - | - | - | - | 3 | 72.0 | 0.1 | 36.0 | 50.0 | 0.2 |
| *Priodontes maximus* | 3 | - | - | - | - | 3 | 72.0 | 0.1 | 36.0 | 50.0 | 0.2 |
| **Perissodactyla** | 25 | 1 | 22 | 1 | 9 | 58 | 8,200.3 | 15.3 | 2,166.2 | 26.4 | 11.7 |
| *Tapirus terrestris* | 25 | 1 | 22 | 1 | 9 | 58 | 8,200.3 | 15.3 | 2,166.2 | 26.4 | 11.7 |
| **Primates** | 292 | 41 | 67 | 6 | 25 | 431 | 2,791.1 | 5.2 | 347.8 | 12.5 | 1.9 |
| *Alouatta seniculus* | 32 | 9 | 7 | - | 3 | 51 | 340.1 | 0.6 | 33.4 | 9.8 | 0.2 |
| *Ateles belzebuth* | 52 | 5 | 11 | 1 | 4 | 73 | 555.7 | 1.0 | 60.7 | 10.9 | 0.3 |
| *Callicebus discolor* | 2 | 3 | - | - | - | 5 | 4.8 | 0.0 | 0.9 | 18.9 | 0.0 |
| *Callithrix pygmaea* | - | - | 1 | - | - | 1 | 0.9 | 0.0 | 0.0 | 0.0 | 0.0 |
| *Cebus albifrons* | 4 | 4 | 1 | - | - | 9 | 35.0 | 0.1 | 2.0 | 5.7 | 0.0 |
| *Lagothrix poeppigii* | 196 | 15 | 47 | 5 | 17 | 280 | 1,838.3 | 3.4 | 249.4 | 13.6 | 1.3 |
| *Pithecia monachus* | 2 | 4 | - | - | - | 6 | 9.9 | 0.0 | 1.5 | 15.1 | 0.0 |
| *Saimiri sciureus* | 4 | 1 | - | - | 1 | 6 | 6.5 | 0.0 | 0.0 | 0.0 | 0.0 |
| **Rodentia** | 99 | 16 | 62 | 8 | 16 | 201 | 1,341.1 | 2.5 | 391.4 | 29.2 | 2.1 |
| *Cuniculus paca* | 51 | 2 | 49 | 2 | 13 | 117 | 1,032.5 | 1.9 | 334.6 | 32.4 | 1.8 |
| *Dasyprocta fuliginosa* | 28 | 3 | 13 | 4 | 1 | 49 | 237.6 | 0.4 | 53.7 | 22.6 | 0.3 |
| *Hydrochoerus hydrochaeris* | - | - | - | - | 1 | 1 | 36.0 | 0.1 | 0.0 | 0.0 | 0.0 |
| *Myoprocta pratti* | 2 | 7 | - | 2 | 1 | 12 | 16.1 | 0.0 | 1.4 | 8.5 | 0.0 |
| *Sciurus* sp. | 8 | 2 | - | - | - | 10 | 8.4 | 0.0 | 1.8 | 21.8 | 0.0 |
| *Sciurus spadiceus* | 10 | 2 | - | - | - | 12 | 10.5 | 0.0 | 0.0 | 0.0 | 0.0 |
| **REPTILIA** | 12 | 1 | 24 | 1 | 4 | 42 | 262.5 | 0.5 | 128.8 | 49.1 | 0.7 |
| **Crocodylia** | 5 | - | 1 | - | 2 | 8 | 82.4 | 0.2 | 7.0 | 8.5 | 0.0 |
| *Caiman crocodylus* | 4 | - | 1 | - | 1 | 6 | 68.4 | 0.1 | 7.0 | 10.2 | 0.0 |
| *Caiman niger* | - | - | - | - | 1 | 1 | 9.0 | 0.0 | 0.0 | 0.0 | 0.0 |
| *Caiman* sp. | 1 | - | - | - | - | 1 | 5.0 | 0.0 | 0.0 | 0.0 | 0.0 |
| **Testudines** | 7 | 1 | 23 | 1 | 2 | 34 | 180.2 | 0.3 | 121.8 | 67.6 | 0.7 |
| *Chelonoidis denticulata* | 7 | 1 | 20 | 1 | 2 | 31 | 168.8 | 0.3 | 110.5 | 65.4 | 0.6 |
| *Podocnemis unifilis* | - | - | 3 | - | - | 3 | 11.4 | 0.0 | 11.4 | 100.0 | 0.1 |
| **TOTAL** | 2,000 | 163 | 635 | 55 | 248 | 3,101 | 53,692.7 | 100.0 | 18,531.9 | 34.5 | 100.0 |

^a^Proportion of Total Biomass: Percentage of species’ contribution to total biomass harvested; ^b^Within Species Trade: Percentage of species’ biomass harvested that is traded; ^c^Proportion of Total Trade: Percentage of species’ contribution to total biomass traded.
