# Supplementary material for: Road Development and the Geography of Hunting by an Amazonian Indigenous Group: Consequences for Wildlife Conservation
Source: PLoS One. 2014 Dec 9;9(12):e114916. doi: 10.1371/journal.pone.0114916 (PMC4260950; doi:10.1371/journal.pone.0114916)
Supplement: S2 Appendix — Threshold-independent receiver operating characteristic (ROC) curve to test predictability performance of model to estimate probability of hunting. (DOCX) [file pone.0114916.s002.docx]

Appendix S2. Threshold-independent receiver operating characteristic (ROC) curve to test predictability performance of model to estimate probability of hunting. Output from SPSS-PASW v.18.

| **Case Processing Summary** | |
| --- | --- |
| kill | Valid N (listwise) |
| Positive^a^ | 2997 |
| Negative | 3000 |
| Larger values of the test result variable(s) indicate stronger evidence for a positive actual state. | |
| ^a^The positive actual state is 1. | |

| **Area Under the Curve** | | | | |
| --- | --- | --- | --- | --- |
| Test Result Variable(s): Predicted Value of Mean of Response | | | | |
| Area | Std. Error^a^ | Asymptotic Sig.^b^ | Asymptotic 95% Confidence Interval | |
|  |  |  | Lower Bound | Upper Bound |
| 0.920 | 0.003 | 0.000 | 0.913 | 0.926 |
| ^a^Under the nonparametric assumption | | | | |
| ^b^Null hypothesis: true area = 0.5 | | | | |
