# Supplementary material for: Road Development and the Geography of Hunting by an Amazonian Indigenous Group: Consequences for Wildlife Conservation
Source: PLoS One. 2014 Dec 9;9(12):e114916. doi: 10.1371/journal.pone.0114916 (PMC4260950; doi:10.1371/journal.pone.0114916)
Supplement: S3 Appendix — Selection of best-fit model with Akaike Information Criterion (AIC). (DOCX) [file pone.0114916.s003.docx]

Appendix S3. Selection of best-fit model with Akaike Information Criterion (AIC).

| Candidate model^*^ | AIC | K | ΔAIC | AIC_w_ |
| --- | --- | --- | --- | --- |
| road + river + settlement | 4197 | 4 | 0 | 1.000 |
| road + river | 4976 | 3 | 779 | 0.000 |
| road | 5968 | 2 | 1772 | 0.000 |
| road + settlement | 5970 | 3 | 1774 | 0.000 |
| river + settlement | 6611 | 3 | 2414 | 0.000 |
| settlement | 6860 | 2 | 2663 | 0.000 |
| river | 7834 | 2 | 3637 | 0.000 |

^*^Candidate models to assess probability of hunting as a function of Euclidean distances (km) to nearest road, river and settlement.
